# History Marginalization Improves Forecasting in Variational Recurrent Neural Networks

**DOI:** 10.3390/e23121563

**Published:** 2021-11-24

**Authors:** Chen Qiu, Stephan Mandt, Maja Rudolph

**Affiliations:** 1 Bosch Center for AI, 71272 Renningen, Germany; Chen.Qiu@de.bosch.com; 2 Department of Computer Science, TU Kaiserslautern, 67653 Kaiserslautern, Germany; 3 Department of Computer Science, University of California, Irvine, CA 92697, USA; Mandt@uci.edu; 4 Bosch Center for AI, Pittsburgh, PA 15222, USA

**Keywords:** sequential latent variable models, time series forecasting, variational inference

## Abstract

Deep probabilistic time series forecasting models have become an integral part of machine learning. While several powerful generative models have been proposed, we provide evidence that their associated inference models are oftentimes too limited and cause the generative model to predict mode-averaged dynamics. Mode-averaging is problematic since many real-world sequences are highly multi-modal, and their averaged dynamics are unphysical (e.g., predicted taxi trajectories might run through buildings on the street map). To better capture multi-modality, we develop variational dynamic mixtures (VDM): a new variational family to infer sequential latent variables. The VDM approximate posterior at each time step is a mixture density network, whose parameters come from propagating multiple samples through a recurrent architecture. This results in an expressive multi-modal posterior approximation. In an empirical study, we show that VDM outperforms competing approaches on highly multi-modal datasets from different domains.

## 1. Introduction

**Figure 1:**
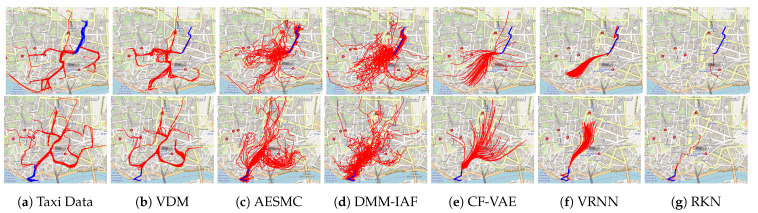
Forecasting taxi trajectories is challenging due to the highly multi-modal nature of the data (**a**). VDM (**b**) succeeds in generating diverse plausible predictions (red), based the beginning of a trajectory (blue). The other methods, auto-encoding sequential Monte Carlo (AESMC) [1], deep Markov model [2] with variational posteriors based on inverse autoregressive flows [3] (DMM-IAF), conditional flow variational autoencoder (CF-VAE) [4], variational recurrent neural network (VRNN) [5], recurrent Kalman network (RKN) [6], suffer from mode averaging.

Making sense of time series data can be challenging, especially in real world data-sets that are highly multi-modal. There may be multiple plausible future projections at any given part of the observed sequence, but the average projection is often highly unlikely or even physically impossible. As an example, consider a dataset of taxi trajectories (https://www.kaggle.com/crailtap/taxi-trajectory, accessed on 1 March 2020). In each row of Figure 1a, we have selected 50 routes from the dataset with similar starting behavior (blue). Even though these routes are quite similar to each other in the first ten waypoints, the continuations of the trajectories (red) can exhibit distinct behaviors and lead to points on any far edge of the map. We see that trajectories follow a few main traffic arteries, which are the data distribution’s modes. Our goal is to learn a generative model of the data that can forecast plausible continuations for the trajectories based on some initial waypoints.

Most data-driven neural forecasting models are based on assumptions such as Gaussianity to make learning tractable and efficient. However, trying to capture the dynamics through unimodal distributions can lead either to “over-generalization” (i.e., placing probability mass in spurious regions) or focusing only on the dominant mode. Even expressive neural approaches based on deep sequential latent variable models fail to capture this multi-modality fully. In this paper, we stress that the shortcomings of these models can be traced back to restrictive modeling assumptions in their approximate *inference*. To address this, we develop variational dynamic mixtures (VDM): a new inference approach for deep sequential latent variable models. Our main contributions are as follows:

- A new inference model. We establish a new type of variational family for inference in sequential latent variable models. Instead of a structured variational approximation, VDM marginalizes over past states. This leads to an efficient mean-field factorization where each variational factor is multi-modal by construction.
- An evaluation metric for multi-modal forecasting. The negative log-likelihood measures predictive accuracy but neglects an important aspect of multi-modal forecasts—sample diversity. In Section 4, we propose a score inspired by the Wasserstein distance [7] which evaluates both prediction quality and diversity. This metric complements our evaluation based on log-likelihoods.
- An extensive empirical study. In Section 4, we use VDM to study various datasets, including synthetic data, a stochastic Lorenz attractor, taxi trajectories, basketball player trajectories, and a U.S. pollution dataset with the measurements of various pollutants over time. We illustrate VDM’s ability in modeling multi-modal dynamics and provide quantitative comparisons to other methods showing that VDM compares favorably to previous work.

## 2. Related Work

Recurrent neural networks (RNNs) such as long short-term memorys (LSTMs) [8] and gated recurrent units (GRUs) [9] have proven successful on many time series modeling tasks. However, as deterministic models they cannot capture uncertainties in their dynamic predictions. Stochastic RNNs make these sequence models non-deterministic [5,10,11,12]. For example, the variational recurrent neural network (VRNN) [5] enables multiple stochastic forecasts due to its stochastic transition dynamics. An extension of VRNN [13] uses an auxiliary cost to alleviate the KL-vanishing problem. It improves on VRNN inference by forcing the latent variables to also be predictive of future observations. Another line of related methods rely on particle filtering [1,14,15] and in particular sequential Monte Carlo (SMC) to improve the evidence lower bound. In contrast, VDM adopts an explicitly multi-modal posterior approximation. Another SMC-based work [16] employs search-based techniques for multi-modality but is limited to models with finite discrete states. Recent works [17,18,19] use normalizing flows in the latent space to model the transition dynamics. A task orthogonal to multi-modal inference is learning disentangled representations. Here too, mixture models are used [20,21]. These papers use discrete variables and a mutual information based term to disentangle different aspects of the data. VAE-like models [4,22] and GAN-like models [23,24] only have global, time independent latent variables. Yet, they show good results on various tasks, including forecasting. With a deterministic decoder, these models focus on average dynamics and do not capture local details (including multi-modal transitions) very well.

Classical state-space models (SSMs) are popular due to their tractable inference and interpretable predictions. Similarly, *deep* SSMs with locally linear transition dynamics enjoy tractable inference [6,25,26,27]. However, these models are often not expressive enough to capture complex (or highly multi-modal) dynamics. Nonlinear deep SSMs [2,28,29,30,31] are more flexible. Their inference is often no longer tractable and requires variational approximations. Unfortunately, in order for the inference to be tractable, the variational approximations are often simplistic and do not approximate multi-modal posteriors well with negative effects on the trained models. Multi-modality can be incorporated via normalizing flows [3] or via additional discrete switching latent variables, such as switching linear dynamical systems [32,33,34].

## 3. Method–Variational Dynamic Mixtures

Variational methods for sequential latent variable models often use a structured posterior approximation [1,2,5,25,26,27,28], where the variational factors condition on past states. These factors are usually considered to be conditional Gaussians. The Gaussian assumption significantly limits the generative model’s dynamics and often leads to mode-averaging behavior. With VDM we develop a variational method for deep sequential latent variable models that overcomes these shortcomings.

Unlike recent work on dynamics modeling, VDM relies on a mean-field assumption. Marginalization over past states mediates temporal dependencies. It has three effects. (1) The factorization of the posterior approximation is mean-field, leading to efficient evidence lower bound (ELBO) computations. (2) The marginalization introduces information about previously inferred dynamics into the variational factors. (3) Each variational factor is a mixture of Gaussians, resulting in an advantageous inference procedure for learning multi-modal dynamics.

We first present the generative model (Section 3.1) and the multi-modal inference model (Section 3.2) of VDM. In Section 3.3, we then present the variational objective including an optional regularization term that gives a nice performance boost. At last, we discuss alternative implementation choices that are optional but can enhance the expressiveness of the model in Section 3.4.

### 3.1. The Generative Model of VDM

Given sequential observations $x_{1:T}=\left(x_{1},\ldots ,x_{T}\right)$, we assume that the underlying dynamics are governed by the latent states $z_{1:T}=\left(z_{1},\ldots ,z_{T}\right)$. Although the approach is more general, we consider a basic deep latent sequence modeling architecture inspired by [5]. The generative modelconsists of a transition and an emission model. The transition model $p(z_{t}|z_{<t})$ describes the temporal evolution of the latent states whose dynamics are governed by a recurrent neural network, such as a GRU [35], $\phi^{\mathrm{GRU}}$ with the hidden state $h_{t}$ [9,28,36] (for a better long term generation, we do not incorporate autoregressive feedback from the data $x_{t}$). The emission model $p(x_{t}|z_{\leq t})$ maps the states to observations. We assume they are parameterized by two separate neural networks, the transition network $\phi^{tra}$ and the emission network $\phi^{dec}$. With $h_{1}$ initialized to a vector of zeros, the latent states $z_{t}$ are sampled recursively as

(1) $$\begin{array}{ccc} \; & p\left(z_{t}|z_{<t}\right)=N\left(\mu_{0,t},\sigma_{0,t}^{2}I\right),\; & \mathrm{where}\;\left[\mu_{0,t},\sigma_{0,t}^{2}\right]=\phi^{tra}\left(h_{t}\right),\;h_{t}=\phi^{\mathrm{GRU}}\left(z_{t-1},h_{t-1}\right). \end{array}$$

Conditioned on $z_{t}$ and $h_{t}$, the data are then generated according to the emission model

(2) $$\begin{array}{ccc} \; & p\left(x_{t}|z_{\leq t}\right)=N\left(\mu_{x,t},\sigma_{x,t}^{2}I\right),\; & \mathrm{where}\;\left[\mu_{x,t},\sigma_{x,t}^{2}\right]=\phi^{dec}\left(z_{t},h_{t}\right). \end{array}$$

Similar generative models have been studied before. The main innovation of VDM is its inference procedure.

### 3.2. The Variational Posterior of VDM

While the VRNN [5] and other variational approaches for neural recurrent models use a structured posterior, we make the mean-field assumption that the variational family factors over time. Even though our generative model is similar to the VRNN, the competitive edge of VDM comes from marginalizing over past states in the inference. Like including an auxiliary variable in the variational factors [37], this makes the posterior approximation more flexible and relates to placing a prior on the variational parameters of the mean-field factors [38,39]. In VDM the past states $z_{<t}$ are treated as auxiliary variables for the marginal posterior at time *t*. This allows the method to pass information about previously inferred dynamics into the variational factors.

(3) $$\begin{array}{c} q\left(z_{1:T}|x_{1:T}\right)=\prod_{t=1}^{T}q\left(z_{t}|x_{\leq t}\right)=\prod_{t=1}^{T}\int q_{\mathrm{aug}}\left(z_{t},z_{<t}|x_{\leq t}\right)dz_{<t}\;. \end{array}$$

While this variational approximation has the added expressiveness of marginalizing out past states, it is mean-field, which leads to advantages when deriving the variational objective. We assume the augmented distribution factorizes into an inference distribution $q_{\inf}$ and a target distribution $q_{\mathrm{tar}}$,

(4) $$\begin{array}{c} q_{\mathrm{aug}}\left(z_{t},z_{<t}|x_{\leq t}\right)=q_{\inf}\left(z_{t}|z_{<t},x_{t}\right)q_{\mathrm{tar}}\left(z_{<t}|x_{\leq t}\right). \end{array}$$

The distributions $q_{\inf}$ and $q_{\mathrm{tar}}$ have different roles:

- $q_{\inf}$ reflects the generative model’s transition dynamics and combines it with the current observation $x_{t}$. It is a Gaussian distribution whose parameters are obtained by propagating $z_{<t}$ through the RNN of the generative model and using an inference network to combine the output with $x_{t}$.
- $q_{\mathrm{tar}}$ is a distribution we will use to sample past states for approximating the marginalization in Equation (3). Its name suggests that it is generally intractable and will be approximated via self-normalized importance sampling.

The variational posterior of VDM marginalizes over past states (Equation (3)). The target distribution specifies how past states are sampled and the inference distribution specifies how the new observation should correct the distribution over latent states. In the simplest version of VDM sampling from the target distribution corresponds to sampling from previous posteriors. Then we show that we can add modeling flexibility by using self-normalized weighted sampling for the target distribution.

#### 3.2.1. Parametrization of the Variational Posterior

The VDM inference approach uses the same RNN as the generative model to track the history of the latent states. By using the RNN to summarize information from past states, sampling from the target distribution can be done efficiently. Using previously inferred posteriors as the target distribution, $q_{\mathrm{tar}}\left(z_{<t}|x_{\leq t}\right):=q\left(z_{<t}|x_{<t}\right)$, past states are sampled sequentially as follows. At each time step *t*, we sample *K* samples from the previous posterior $z_{t-1}^{\left(i\right)}∼q\left(z_{t-1}|x_{<t}\right)$ indexed by *i* and these samples are aggregated by the RNN (with same parameters $\phi^{\mathrm{GRU}}$ as in the generative model.)

(5) $$z_{t-1}^{\left(i\right)}∼q\left(z_{t-1}|x_{<t}\right)\;h_{t}^{\left(i\right)}=\phi^{\mathrm{GRU}}\left(z_{t-1}^{\left(i\right)},{\hat{h}}_{t-1}\right),\;{\hat{h}}_{t}=E_{q_{\mathrm{tar}}\left(z_{<t}|x_{\leq t}\right)}\left[h_{t}\right]\;.$$

We initialize $\hat{h_{1}}$ to a vector of zeros. To avoid an exponential blow-up of the number of samples as *t* increases, we compute an expected history ${\hat{h}}_{t}$ for the recursion of the RNN.

To evaluate the inference distribution on each of the samples, an inference network $\phi^{inf}$ combines the output of the RNN with the new observation $x_{t}$ to produce the mean and variance of $q_{\inf}$ that we assume to be Gaussian

(6) $$\begin{array}{c} q_{\inf}\left(z_{t}|z_{<t}^{\left(i\right)},x_{t}\right)=N\left(\mu_{\inf,t}^{\left(i\right)},\sigma_{\inf,t}^{(i)2}I\right),\;\mathrm{where}\;\left[\mu_{\inf,t}^{\left(i\right)},\sigma_{\inf,t}^{(i)2}\right]=\phi^{inf}\left(h_{t}^{\left(i\right)},x_{t}\right)\;. \end{array}$$

We use the notation $z_{<t}^{\left(i\right)}$ to indicate that the parameters of the distribution are computed as a function of $h_{t}^{\left(i\right)}$ as defined in Equation (5). By using the transition dynamics of the generative model, the inference model can focus its capacity on learning how to account for the new observation when inferring $z_{t}$. Given samples from the target distribution, we can approximate the marginalization in Equation (3) to obtain

(7) $$\begin{array}{cc} q(z_{t}|x_{\leq t}) & =E_{q_{\mathrm{tar}}\left(z_{<t}|x_{\leq t}\right)}\left[q_{\inf}\left(z_{t}|z_{<t},x_{t}\right)\right]\\ \; & \approx \sum_{i=1}^{K}\omega_{t}^{\left(i\right)}q_{\inf}\left(z_{t}|z_{<t}^{\left(i\right)},x_{t}\right)=\sum_{i=1}^{K}\omega_{t}^{\left(i\right)}N\left(\mu_{\inf,t}^{\left(i\right)},\sigma_{\inf,t}^{(i)2}I\right),\;\mathrm{where}\;\omega_{t}^{\left(i\right)}=\frac{1}{K}. \end{array}$$

The marginal variational posterior becomes an equally weighted mixture density network [40], which is a good choice for modeling multi-modal dynamics (as our experiments show). The variational posterior of VDM can gain additional modeling flexibility by choosing different parametrizations for the mixture weights.

#### 3.2.2. Generalized Mixture Weights

Assume that we chose a target distribution that is different from the past approximate posterior $q_{\mathrm{tar}}\left(z_{<t}|x_{\leq t}\right)\neq q\left(z_{<t}|x_{<t}\right)$. If we still use samples from the past posterior to approximate the marginalization in Equation (7), the *importance weights* $\omega_{t}^{\left(i\right)}$ have to correct for the discrepancy between the base distribution (approximate posterior) and the target distribution $q_{\mathrm{tar}}$ ([41], Ch. 9). In a more general variational family for VDM than described above, the target distribution does not equal the base distribution. In this generalized setting, instead of choosing a parametrization for $q_{\mathrm{tar}}$ and then deriving the importance weights, we directly choose how to parameterize the weights which we ensure are self-normalized ([41], Ch. 9.2). We choose the generalized weights to be,

(8) $$\begin{array}{cc} \; & \omega_{t}^{\left(i\right)}:=\omega \left(x_{t},z_{<t}^{\left(i\right)}\right)/\left(\sum_{j=1}^{K}\omega \left(x_{t},z_{<t}^{\left(j\right)}\right)\right), \end{array}$$

(9) $$\begin{array}{cc} \; & \mathrm{where}\;\;\omega \left(x_{t},z_{<t}\right):=p\left(x_{t}|z_{<t}\right)=E_{z_{t}}\left[p\left(x_{t}|z_{\leq t}\right)p\left(z_{t}|z_{<t}\right)\right]. \end{array}$$

With this definition the weights are normalized by construction, $\sum_{i=1}^{K}\omega_{t}^{\left(i\right)}=1$. We could choose any finite and non-negative expression for $\omega (x_{t},z_{<t})$. As in importance sampling for bootstrap particle filters [42], our choice of weights takes into account each sample’s relevance for predicting the new observation $x_{t}$. Another advantage is that we do not introduce additional variational parameters. The only variational parameters of the VDM inference model are the neural network parameters of $\phi^{inf}$. The predictive likelihood $p(x_{t}|z_{\leq t})$, can be computed by plugging in samples $z_{<t}^{\left(i\right)}$, that are sampled and aggregated according to Equation (5), into the generative model (Equations (1) and (2)). Pseudo code for the generative and the inference model are in Algorithms 1 and 2.
**Algorithm 1:** Generative model.
 **Inputs:**
$z_{\tau },h_{\tau }$
 **Outputs:**
$x_{\tau +1:T}$
 **for**
t=τ+1:T
**do**
  $h_{t}=\phi^{\mathrm{GRU}}\left(z_{t-1},h_{t-1}\right)$
  $\left[\mu_{0,t},\sigma_{0,t}^{2}\right]=\phi^{tra}\left(h_{t}\right)$ Equation (1)
  $z_{t}∼N\left(\mu_{0,t},\sigma_{0,t}^{2}I\right)$
  $\left[\mu_{x,t},\sigma_{x,t}^{2}\right]=\phi^{dec}\left(z_{t},h_{t}\right)$ Equation (2)
  $x_{t}∼N\left(\mu_{x,t},\sigma_{x,t}^{2}I\right)$
 **end for**


**Algorithm 2:** Inference model.
 **Inputs:**
$x_{1:\tau },{\hat{h}}_{1}$
 **Outputs:**
$z_{1:\tau },{\hat{h}}_{\tau }$
 $\left[\mu_{\inf,1},\sigma_{\inf,1}^{2}\right]=\phi^{inf}\left(x_{1},{\hat{h}}_{1}\right)$
 $z_{1}^{\left(i\right)}∼N\left(\mu_{z,1},\sigma_{z,1}^{2}I\right)$
 **for**
t=2:τ
**do**
  $h_{t}^{\left(i\right)}=\phi^{\mathrm{GRU}}\left(z_{t-1}^{\left(i\right)},{\hat{h}}_{t-1}\right)$ Equation (5)
  $\left[\mu_{\inf,t}^{\left(i\right)},\sigma_{\inf,t}^{(i)2}\right]=\phi^{inf}\left(x_{t},h_{t}^{\left(i\right)}\right)$ Equation (6)
  $\omega_{t}^{\left(i\right)}\omega \left(x_{t},h_{t}^{\left(i\right)}\right)/\sum_{j=1}^{K}\omega \left(x_{t},h_{t}^{\left(j\right)}\right)$ Equation (8)
  $z_{t}^{\left(i\right)}∼\sum_{i}^{k}\omega_{t}^{\left(i\right)}N\left(\mu_{\inf,t}^{\left(i\right)},\sigma_{\inf,t}^{(i)2}I\right)$
  ${\hat{h}}_{t}=\sum_{i}^{k}\omega_{t}^{\left(i\right)}h_{t}^{\left(i\right)}$
 **end for**


It is interesting to wonder about the connections to structured variational inference. If we do not marginalize over $z_{<t}$ but rather condition on it (use the same inference distribution $q_{\inf}$), we obtain the structured variational approximation used in the conventional VRNN approach. The advantage of instead carrying out the marginalization is that we explore multiple modes of the transition dynamics. Approximating the marginalization in Equation (3) with a single sample (K=1), recovers the inference model of VRNN [5].

### 3.3. The Variational Objective of VDM

VDM is fit with a variational objective. It consists of the ELBO terms and an optional regularization term that is helpful to improve the performance. In our empirical study, we investigate the effect of the regularization term both for VDM and for other existing methods. We found that when the method worked well without the regularization term, the regularization term gave an additional performance boost, especially on the qualitative results.

We will first describe the ELBO for VDM and then motivate and explain the regularization term. As in [37] the ELBO is derived based on the augmented model in Equation (4). The main challenge is to lower-bound the entropy of the augmented variational distribution, which contains an implicit component. In Appendix A, we show that this quantity can be lower-bounded and that the lower bound can be estimated using the reparameterization trick. The resulting instantaneous ELBO is:

(10) $$\begin{array}{cc} \; & \log p\left(x_{1:T}\right)\geq L_{\mathrm{ELBO}}\\ \; & =\sum_{t=1}^{T}\sum_{i=1}^{K}\omega_{t}^{\left(i\right)}E_{q_{\inf}\left(z_{t}|z_{<t}^{\left(i\right)},x_{t}\right)}\left[\log p\left(x_{t}|z_{t},z_{<t}^{\left(i\right)}\right)-\log\left(\sum_{i=1}^{K}\omega_{t}^{\left(i\right)}q_{\inf}\left(z_{t}|z_{<t}^{\left(i\right)},x_{t}\right)\right)\right]\\ \; & +E_{q(z_{1}|x_{1})}\left[\log p(z_{1})\right]+\sum_{t=2}^{T}\sum_{i=1}^{K}\omega_{t}^{\left(i\right)}E_{q_{\inf}\left(z_{t}|z_{<t}^{\left(i\right)},x_{t}\right)}\left[\log p(z_{t}|z_{<t}^{\left(i\right)})\right]\; \end{array}$$

Given a dataset D, VDM’s parameters of the generative and inference model $\phi =[\phi^{tra},\phi^{dec},\phi^{\mathrm{GRU}},\phi^{inf}]$ are obtained by minimizing the loss

(11) $$\begin{array}{c} L_{\mathrm{VDM}}\left(\phi \right)=E_{D}\left[-L_{\mathrm{ELBO}}\left(\phi \right)-\sum_{t=1}^{T}\lambda L_{pred,t}\left(\phi \right)\right]\;, \end{array}$$

with a hyperparameter λ determining the strength of the regularization. We propose to augment the ELBO with a *prediction term*. We empirically compare the effect of including and excluding the regularization term in the objective. VDM is competitive without the prediction term, but we got the strongest when including the regularization term $L_{pred,t}$. We set the hyper-parameter λ=1, though an additional performance boost could be obtained by tuning it on the validation set.

The prediction term $L_{pred}$, encourages the variational posterior (from the previous time step) to produce samples that maximize the predictive likelihood,

(12) $$\begin{array}{c} L_{pred,t}\left(\phi \right)=\log E_{q(z_{t-1}|x_{<t})}\left[p(x_{t}|z_{<t})\right]\approx \log\frac{1}{K}\sum_{i}^{K}p\left(x_{t}|z_{t-1}^{\left(i\right)},h_{t-1}\right)\;, \end{array}$$

the likelihood under each sample $p(x_{t}|z_{t-1}^{\left(i\right)},h_{t-1})$ is assumed to be Gaussian. The mean and variance of this distribution are computed by propagating the sample through the transition model (Equation (1)) and the result through the emission model (Equation (2)) (see Algorithm 1.) This regularization term is helpful to improve the prediction performance since it depends on the predictive likelihood of samples, which is not involved in the ELBO.

### 3.4. Alternative Modeling Choices

Next, we discuss alternative implementations of VDM that are optional, but can enhance the expressiveness of the model.

Our method involves sampling from Gaussian distributions at multiple steps. While Monte-Carlo (MC) methods work, it turns out that we can achieve better results with fewer samples by drawing on so-called cubature approximations [43,44,45], which choose samples more carefully. In our stochastic cubature approximation (SCA), the usually deterministically-chosen curbature points are further randomized for better performance, allowing us to use fewer samples than in naive MC. See Appendix B for more details.

An alternative choice of the expression for the weights is

(13) $$\begin{array}{c} \omega \left(x_{t},z_{<t}^{\left(i\right)}\right):=1\left(i=\underset{j\in [1,\cdots ,K]}{arg max}p\left(x_{t}|z_{<t}^{\left(j\right)}\right)\right). \end{array}$$

which corresponds to a hard choice between the samples. Only the component associated with the sample that achieves the highest predictive likelihood is nonzero. We stress that this choice for the weights still corresponds to a multi-modal posterior approximation: all *K* mixture components that result from propagating different latent states $z_{<t}^{\left(j\right)}$ through the GRU are considered as candidate modes, and the most likely mixture component is selected after new data is observed. Even though each single observation is assigned only to a single mode, the combination of the modes (namely a mixture) is used to model the entire data. Similarly as in “best-of-many” sampling [22], the zeroed-out components in the mixture density network have the capacity to focus on other modes. We found the hard choice works well in our empirical study and use it as the default choice for VDM.

## 4. Evaluation and Experiments

In this section, we evaluate VDM’s ability to model multi-modal dynamics and show its competitive forecasting performance in various domains. We first introduce the evaluation metrics, baselines and summarize all ablations. Experiments on synthetic data demonstrate that VDM is truly multi-modal thereby supporting the modeling choices of Section 3, especially for the inference model. Experiments on real-world datasets with challenging multi-modal dynamics show the benefit of VDM over state-of-the-art (deep) probabilistic time-series models.

### 4.1. Evaluation Metrics

In the experiments, we create a training set, a validation set, and a test set. During validation and test, each trajectory is split into two parts; initial observations (given to the models for inference) and continuations of the trajectory (to be predicted and not accessible to the models). The inference models are used to process the initial observations and to infer latent states. These are then processed by the generative models to produce forecasts.

We use 3 criteria to evaluate these forecasts (i) multi-step prediction $p(x_{t+1:t+\tau }|x_{1:t})$, (ii) one-step-ahead prediction $p(x_{t+1}|x_{1:t})$, and (iii) a new metric inspired by the Wasserstein distance. As in other work [4,22,46], (i) and (ii) are reported in terms of negative log-likelihood. When the models’ predictive distribution for one-step-ahead prediction is assumed to be Gaussian, its negative log-likelihood can be computed in closed form. However, the long-term forecasts have to be evaluated using samples. For each ground truth x we generate n=1000 forecasts ${\hat{x}}_{i}$ given initial observations from the beginning. For a fair comparison with methods that do not output a predictive variance, we choose a constant variance.

(14) $$NLL=-\log\left(\frac{1}{n}\sum_{i}^{n}\frac{1}{\sqrt{2\pi }}\exp\left(-\frac{{\left({\hat{x}}_{i}-x\right)}^{2}}{2}\right)\right)\;.$$

This evaluates the predictive accuracy but neglects a key aspect of multi-modal forecasts–diversity. We propose a new evaluation metric, which takes both diversity and accuracy of predictions into account. Inspired by the Wasserstein distance [7], we compute the distance between the ground truth distribution X and the model distribution $\hat{X}$ as

(15) $$W\left(X,\hat{X}\right)=\underset{\pi }{\inf}\left(\frac{1}{n}\sum_{i}^{n}∥{(x_{i}-{\hat{x}}_{\pi (i)}∥}_{2}\right)\;,$$

where x and $\hat{x}$ are the ground truth sequences and model forecasts, and π denotes all permutations. We select *n* samples from the test set with similar initial observations. The model is expected to generate samples matching all ground truth continuations given the initial observations. The model generates 10×n forecasts. We compute the distance between *n* ground truth sequences and the top *n* well-matched predictions with Equation (15). Since the forecasts do not match with ground truth sequences one to one well due to the randomness, we generate more forecasts to mitigate the variance of the results. We report the average of W-distances over different initial observations.

### 4.2. Baselines

We choose baselines from three classes of models. Two stochastic recurrent models are variational recurrent neural network (VRNN) [5] and auto-encoding sequential Monte Carlo (AESMC) [1]. VRNN has a similar but more powerful generative model than VDM, and AESMC uses SMC to achieve a tighter lower bound. However, compared to VDM, both use the structured variational approximation rather than marginalizing over past states. Two deep SSMs are recurrent Kalman network (RKN) [6] and deep Markov model [2] with variational posteriors based on inverse autoregressive flows [3] (DMM-IAF). RKN models the latent space with locally linear SSMs. DMM-IAF is a nonlinear deep SSM leveraging a structured variational inference with flexible variational distributions based on flows. A final baseline is conditional flow variational autoencoder (CF-VAE) [4], a global latent variable model based on normalizing flows.

For fair comparisons, we add recurrent states to DMM-IAF, and fix the dimension of the latent variables $z_{t}$ and $h_{t}$ to be the same for VDM, AESMC, DMM-IAF and VRNN which have the same resulting model size (except for the additional autoregressive feedback in VRNN, and additional flows in DMM-IAF). AESMC and VDM use the same number of samples. RKN does not have recurrent states, so we choose a higher latent dimension to make model size comparable. CF-VAE has only one global latent variable which needs more capacity and we make it higher-dimensional than $z_{t}$. Implementation details are in Appendix D. Since $L_{pred}$ can be easily applied to all baselines except for CF-VAE, we trained them with or without $L_{pred}$, and report the best results.

### 4.3. Ablations

VDM has many ingredients; the type of sampling method, different approximation schemes for the expectations w.r.t. $q_{\mathrm{tar}}$, and the optional regularization term, which can also be beneficial to existing methods. To disentangle the contributions of the varying ingredients we include an extensive ablation study. The definition of all VDM variants is in Table 1. VDM is the default model using improved Gaussian sampling, *hard* weights for the mixtures (Equation (13)), and trained with $L_{\mathrm{VDM}}$. In VDM ($L_{\mathrm{ELBO}}$), we study the contribution of the prediction term and only use $L_{\mathrm{ELBO}}$ as the training objective. In VDM-SCA-S, we use improved Gaussian sampling and *soft* weights (Equation (9)) instead, in VDM-MC-S, we use Monte-Carlo sampling and soft weights, while in VDM-MC-U, we use Monte-Carlo sampling and uniform weights ($\omega_{t}^{\left(i\right)}=\frac{1}{K}$). The comparison of them allows us to understand the effect of different modeling choices: various VDM variants typically outperform the sequential latent variable baselines, and the fine-tuned modeling choices provide a performance boost (shown in Table 2 and Table 3).

### 4.4. Results

We evaluate VDM on synthetic data and three real-world datasets: taxi trajectories, NBA SportVu data, and U.S. pollution data. The experiment on synthetic data demonstrates that VDM is truly multi-modal. By comparing with existing methods on real-world datasets, we show the benefit of VDM over state-of-the-art (deep) probabilistic time-series models.

#### 4.4.1. Synthetic Data with Multi-Modal Dynamics

**Figure 2:**
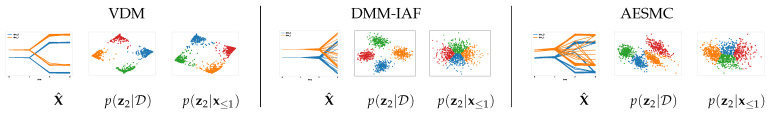
Experiments on 2d synthetic data with 4 modes highlight the multi-modality of VDM. We train VDM (left), DMM-IAF (middle), and AESMC (right) on a training set of trajectories D of length 4, and plot generated trajectories $\hat{X}$ (2 colors for 2 dimensions). VDM and AESMC both use 9 samples. We also plot the aggregated posterior $p(z_{2}|D)$, and the predictive prior $p(z_{2}|x_{\leq 1})$ (4 colors for 4 clusters, and not related to the colors in the trajectories plot) at the second time step. Only VDM learns a multi-modal predictive prior, which explains its success in modeling multi-modal dynamics.

We generate synthetic data with two dimensions and four modes and compare the performance of VDM with 9 samples (Figure 2, left), DMM-IAF (Figure 2, middle), and AESMC using 9 particles (Figure 2, right). Since variational inference is known to try to match the aggregated posterior with the predictive prior [47], it is instructive to fit all three models and to look at their predictive prior $p(z_{2}|x_{\leq 1})$ and the aggregated posterior $p(z_{2}|D)$. Because of the multi-modal nature of the problem, all 3 aggregated posteriors are multi-modal, but only VDM (K=9) learns a multi-modal predictive prior (thanks to its choice of the variational family). Although AESMC and DMM-IAF with flexible structured variational distributions achieve a good match between the prior and the aggregated posterior, the predictive prior does not clearly separate into different modes. In contrast, the inference model of VDM successfully uses multiple samples and explores multiple modes of the transition dynamics to separate latent states into separate modes.

#### 4.4.2. Stochastic Lorenz Attractor

**Figure 3:**
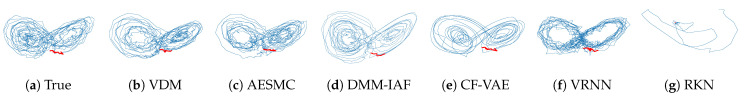
Generated samples from VDM and baselines for stochastic Lorenz attractor. The models generate the future 990 steps (blue) based on the first 10 observations (red). Due to the chaotic property, the reconstruction is impossible even the model learns the right dynamics. VDM, AESMC, and DMM-IAF capture the stochastic dynamics well, while RKN fails.

The Lorenz attractor is a deterministic system governed by ordinary differential equations. Under certain parameter settings, it is chaotic—even small errors can cause considerable differences in the future. We add noise to the transition and emission function to make it stochastic (details in Appendix C). All models are trained and then tasked to predict 90 future observations given 10 initial observations. Figure 3 illustrates qualitatively that VDM (Figure 3b), AESMC (Figure 3c), and DMM-IAF (Figure 3d) succeed in modeling the chaotic dynamics of stochastic Lorenz attractor, while CF-VAE (Figure 3e) and VRNN (Figure 3f) miss local details, and RKN (Figure 3g) which lacks the capacity for stochastic transitions does not work at all. In terms of quantitative results, VDM achieves the best scores on multi-step prediction and W-distance, while VDM-MC-S works best on one-step prediction (Table 2). VDM ($L_{\mathrm{ELBO}}$) does not include $L_{pred}$ in the training and is therefore outperformed by other VDM variants. The baselines AESMC and DMM-IAF also give comparable results. Since the dynamics of Lorenz attractor are governed by ordinary differential equations, the transition dynamics at each time step are not obviously multi-modal, which explains why all models with stochastic transitions do reasonably well. Next, we will show the advantages of VDM on real-world data with multi-modal dynamics.

#### 4.4.3. Taxi Trajectories

**Figure 4:**
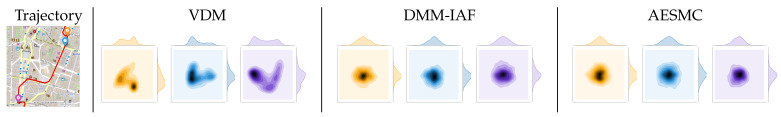
An illustration of predictive priors $p(z_{t}|x_{<t})$ of taxi trajectories from VDM, DMM-IAF, and AESMC at 3 forks in the road marked on the map. VDM and AESMC both use 13 samples. VDM succeeds in capturing the multi-modal distributions, while DMM-IAF and AESMC approximate them with uni-modal distributions. For visualization, the distributions are projected to 2d with KDE.

The taxi trajectory dataset involves taxi trajectories in Porto, Portugal. Each trajectory is a sequence of two-dimensional locations over time. Here, we cut the trajectories to a fixed length of 30 to simplify the comparison (details in Appendix C). The task is to predict the next 20 observations given 10 initial observations. Ideally, the forecasts should follow the street map (though the map is not accessible to the models). The results in Table 2 show that VDM variants typically outperform the other *sequential* latent variable models quantitatively. By tuning the modeling choices of sampling, weights, and the objective, VDM achieves the best results on the one-step prediction and W-distance that measures both diversity and accuracy of predictions. CF-VAE which is a global latent variable model, achieves the lowest negative log-likelihood in multi-step prediction. However, this value does not match the qualitative results in Figure 1. Since CF-VAE has to encode the entire structure of the trajectory forecast into a single latent variable, its predictions seem to average over plausible continuations but are locally neither plausible nor accurate. In comparison, VDM and the other models involve a sequence of latent variables. As the forecasting progresses, the impact of the initial observations becomes weaker and weaker. As a result, local structure can be captured more accurately. While the forecasts are plausible and can be highly diverse, they potentially evolve into other directions than the ground truth. For this reason, their multi-step prediction results are worse in terms of log-likelihood. That is why the empirical W-distance is useful to complement the evaluation of multi-modal tasks. It reflects that the forecasts of VDM are diverse and plausible. Additionally, we illustrate the predictive prior $p(z_{t}|x_{<t})$ at different time steps in Figure 4. VDM learns a multi-modal predictive prior, while AESMC and DMM-IAF result in an uni-modal predictive prior, even though they employ flexible variational distributions.

#### 4.4.4. NBA SportVu Data

**Figure 5:**
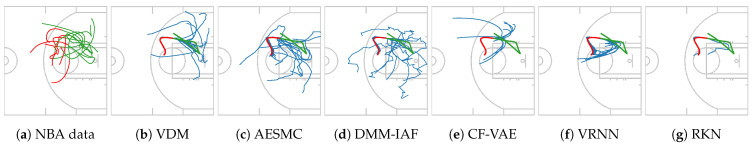
VDM and CF-VAE generate plausible multi-modal trajectories of basketball plays. Each model’s forecasts (blue) are based on the first 10 observations (red). Ground truth data is green.

This dataset (A version of the dataset is available at https://www.stats.com/data-science/, accessed on 1 September 2020) consists of sequences of 2D coordinates describes the movements of basketball players and the ball. We extract the trajectories and cut them to a fixed length of 30 to simplify the comparisons (details in Appendix C). The task is to predict the next 20 observations given 10 initial observations. Players can move anywhere on the court and hence their movement is less structured than taxi trajectories that are constrained by the underlying street map. Due to this, the initial movement patterns are not similar enough to evaluate W-distance. VDM outperforms all baselines and other VDM variants in the multi-step prediction and one-step prediction (Table 3). Other VDM variants perform also reasonably well and better than the other *sequential* latent variable models. Figure 5 illustrates qualitatively that VDM (Figure 5b) and CF-VAE (Figure 5e) succeed in capturing the multi-modal dynamics. The forecasts of AESMC (Figure 5c) and DMM-IAF (Figure 5d) are less plausible (not as smooth as data). VRNN (Figure 5f) and RKN (Figure 5g) fail in capturing the multi-modality.

#### 4.4.5. U.S. Pollution Data

(https://www.kaggle.com/sogun3/uspollution, accessed on 1 March 2020). In this experiment, we study VDM on the U.S. pollution dataset (details in Appendix C). The data is collected from counties in different states from 2000 to 2016. Each observation has 12 dimensions (mean, max value, and air quality index of NO_2_, O_3_, SO_2_, and CO). The goal is to predict monthly pollution values for the coming 18 months, given observations of the previous 6 months. We ignore the geographical location and time information to treat the development tendency of pollution in different counties and different times as i.i.d. The unknown context information makes the dynamics multi-modal and challenging to predict accurately. Due to the small size and high dimensionality of the dataset, there are not enough samples with very similar initial observations. Thus, we cannot evaluate empirical W-distance in this experiment. VDM outperforms all baselines in both evaluations (Table 3).

## 5. Conclusions

We presented variational dynamic mixtures (VDM), a new approach to inference in sequential latent variable models that improves the model’s ability to forecast multi-modal dynamics. The main ideas of VDM is a mean-field factorization with history marginalization, which introduces more complete information about previously inferred dynamics into the variational factors. We also promoted the Wasserstein-distance like metric to evaluate multi-modal forecasting tasks. VDM succeeds in learning challenging multi-modal dynamics and outperforms existing methods on a variety of data sets.

## Figures and Tables

**Table 1:** Definition of VDM variants. By tuning the modeling choices of sampling (MC sampling or SCA in Section 3.4), weights (uniform weights, soft weights in Equation (9), or hard weights in Equation (13)), and the loss function (with or without $L_{pred}$), we propose 5 variants of VDM.

	VDM	VDM ($L_{\mathrm{ELBO}}$)	VDM-SCA-S	VDM-MC-S	VDM-MC-U
Sampling	SCA	SCA	SCA	Monte-Carlo	Monte-Carlo
Weights	hard	hard	soft	soft	uniform
Loss	$L_{\mathrm{VDM}}$	$-L_{\mathrm{ELBO}}$	$L_{\mathrm{VDM}}$	$L_{\mathrm{VDM}}$	$L_{\mathrm{VDM}}$

**Table 2:** Prediction error on stochastic Lorenz attractor and taxi trajectories for three evaluation metrics (details in main text). On the stochastic Lorenz attractor, VDM achieves the best performance. AESMC and DMM-IAF also give comparable results. On the taxi trajectories, CF-VAE achieves the best result in multi-step ahead prediction, since it uses a global variable, that guides the trajectories into generally the right direction. Meanwhile VDM variants outperform all sequential models, and outperform CF-VAE on the other metrics. To test different modeling choices we include the VDM variants of Table 1.

	Stochastic Lorenz Attractor			Taxi Trajectories		
	Multi-Step	One-Step	W-Distance	Multi-Step	One-Step	W-Distance
RKN	104.41	1.88	16.16	4.25	−2.90	2.07
VRNN	65.89 ± 0.21	−1.63	16.14 ± 0.006	5.51 ± 0.002	−2.77	2.43 ± 0.0002
CF-VAE	32.41 ± 0.13	n.a	8.44 ± 0.005	**2.77 ± 0.001**	n.a	0.76 ± 0.0003
DMM-IAF	25.26 ± 0.24	−1.29	7.47 ± 0.014	3.29 ± 0.001	−2.45	0.70 ± 0.0003
AESMC	25.01 ± 0.22	−1.69	**7.29 ± 0.005**	3.31 ± 0.001	−2.87	0.66 ± 0.0004
VDM	**24.49 ± 0.16**	−1.81	**7.29 ± 0.003**	2.88 ± 0.002	**−3.68**	**0.56 ± 0.0008**
$\mathrm{VDM}(L_{\mathrm{ELBO}})$	25.01 ± 0.27	−1.74	7.30 ± 0.004	3.10 ± 0.005	−3.05	0.61 ± 0.0003
VDM−SCA−S	24.69 ± 0.16	−1.83	7.30 ± 0.009	3.09 ± 0.001	−3.24	0.64 ± 0.0005
VDM−MC−S	24.67 ± 0.16	**−1.84**	7.30 ± 0.005	3.17 ± 0.001	−3.21	0.68 ± 0.0008
VDM−MC−U	25.04 ± 0.28	−1.81	7.31 ± 0.002	3.30 ± 0.002	−2.42	0.69 ± 0.0002

**Table 3:** Prediction error on basketball players’ trajectories and U.S. pollution data for two evaluation metrics (details in main text). VDM makes the most accurate multi-step and one-step ahead predictions. The tested variants of VDM are defined in Table 1.

	NBA SportVu		US Pollution	
	Multi-Steps	One-Step	Multi-Steps	One-Step
RKN	4.88	1.55	53.13	6.98
VRNN	5.42 ± 0.009	−2.78	49.32 ± 0.13	8.69
CF-VAE	3.24 ± 0.003	n.a	45.86 ± 0.04	n.a
DMM-IAF	3.63 ± 0.002	−3.74	44.82 ± 0.11	9.41
AESMC	3.74 ± 0.003	−3.91	41.14 ± 0.13	6.93
VDM	**3.23 ± 0.003**	**−5.44**	**37.64 ± 0.07**	**6.91**
$\mathrm{VDM}(L_{\mathrm{ELBO}})$	3.29 ± 0.003	−5.04	39.87 ± 0.04	7.60
VDM−SCA−S	3.31 ± 0.001	−5.08	39.58 ± 0.09	7.82
VDM−MC−S	3.35 ± 0.007	−5.00	40.33 ± 0.03	8.12
VDM−MC−U	3.39 ± 0.006	−4.82	41.81 ± 0.10	7.71

## Data Availability

Not applicable.

## Appendix A. ELBO Derivations

The generative model is

(A1) $$\begin{array}{c} p\left(z_{1:T},x_{1:T}\right)=p\left(z_{1}\right)\prod_{t=2}^{T}p\left(z_{t}|z_{<t}\right)\prod_{t=1}^{T}p\left(x_{t}|z_{\leq t}\right)\;. \end{array}$$

The inference model is

(A2) $$\begin{array}{c} q\left(z_{1:T}|x_{1:T}\right)=\prod_{t=1}^{T}q\left(z_{t}|x_{\leq t}\right)=\prod_{t=1}^{T}\int q_{\inf}\left(z_{t}|z_{<t},x_{t}\right)q_{\mathrm{tar}}\left(z_{<t}|x_{\leq t}\right)dz_{<t}. \end{array}$$

The KL divergence between the approximate posterior and the true posterior of $z_{1:T}$ is

(A3) $$\begin{array}{c} \mathrm{KL}\left(q\left(z_{1:T}|x_{1:T}\right)||p\left(z_{1:T}|x_{1:T}\right)\right)=E_{q(z_{1:T}|x_{1:T})}\left[\log q\left(z_{1:T}|x_{1:T}\right)-\log\frac{p(z_{1:T},x_{1:T})}{p(x_{1:T})}\right] \end{array}$$

and since the KL divergence is non-negative we get the following evidence lower bound

(A4) $$\begin{array}{cc} \; & \log p(x_{1:T})\geq \\ \; & E_{q(z_{1:T}|x_{1:T})}\left[\sum_{t=1}^{T}\log p\left(x_{t}|z_{\leq t}\right)+\log p\left(z_{1}\right)+\sum_{t=2}^{T}\log p\left(z_{t}|z_{<t}\right)-\log q\left(z_{1:T}|x_{1:T}\right)\right]\;. \end{array}$$

We derive each of the three terms (the reconstruction, the cross-entropy, and the entropy) separately. This is the derivation of the reconstruction term:

$$\begin{array}{cc} \; & E_{q(z_{1:T}|x_{1:T})}\left[\sum_{t=1}^{T}\log p\left(x_{t}|z_{\leq t}\right)\right] \end{array}$$

(A5) $$\begin{array}{cc} \; & =\sum_{t=1}^{T}\int q_{\mathrm{aug}}\left(z_{\leq t}|x_{\leq t}\right)\left[\log p(x_{t}|z_{t},z_{<t})\right]dz_{\leq t} \end{array}$$

(A6) $$\begin{array}{cc} \; & =\sum_{t=1}^{T}\;\int \int \;q_{\inf}\left(z_{t}|z_{<t},x_{t}\right)q_{\mathrm{tar}}\left(z_{<t}|x_{\leq t}\right)\left[\log p(x_{t}|z_{t},z_{<t})\right]dz_{<t}dz_{t} \end{array}$$

(A7) $$\begin{array}{cc} \; & \approx \sum_{t=1}^{T}\int \sum_{i=1}^{K}\omega_{t}^{\left(i\right)}q_{\inf}\left(z_{t}|z_{<t}^{\left(i\right)},x_{t}\right)\log p\left(x_{t}|z_{t},z_{<t}^{\left(i\right)}\right)dz_{t} \end{array}$$

(A8) $$\begin{array}{cc} \; & =\sum_{t=1}^{T}\sum_{i=1}^{K}\omega_{t}^{\left(i\right)}E_{q_{\inf}\left(z_{t}|z_{<t}^{\left(i\right)},x_{t}\right)}\left[\log p(x_{t}|z_{t},z_{<t}^{\left(i\right)})\right]\;, \end{array}$$

In Equations (A5) and (A6) we have used the definition of the approximate posterior from Equations (3) and (4), and in Equation (A7) we approximate the integration over the target distribution with samples as defined in Section 3.2.

This is the derivation of the negative cross-entropy term:

$$\begin{array}{cc} \; & E_{q(z_{1:T}|x_{1:T})}\left[\log p\left(z_{1}\right)+\sum_{t=2}^{T}\log p\left(z_{t}|z_{<t}\right)\right] \end{array}$$

(A9) $$\begin{array}{cc} \; & =E_{q(z_{1}|x_{1})}\left[\log p(z_{1})\right]+\sum_{t=2}^{T}\int q_{\mathrm{aug}}\left(z_{\leq t}|x_{\leq t}\right)\left[\log p(z_{t}|z_{<t})\right]dz_{\leq t} \end{array}$$

(A10) $$\begin{array}{cc} \; & =E_{q(z_{1}|x_{1})}\left[\log p(z_{1})\right]+\sum_{t=2}^{T}\;\int \int \;q_{\inf}\left(z_{t}|z_{<t},x_{t}\right)q_{\mathrm{tar}}\left(z_{<t}|x_{\leq t}\right)\left[\log p(z_{t}|z_{<t})\right]dz_{<t}dz_{t} \end{array}$$

(A11) $$\begin{array}{cc} \; & \approx E_{q(z_{1}|x_{1})}\left[\log p(z_{1})\right]+\sum_{t=2}^{T}\sum_{i=1}^{K}\omega_{t}^{\left(i\right)}E_{q_{\inf}\left(z_{t}|z_{<t}^{\left(i\right)},x_{t}\right)}\left[\log p(z_{t}|z_{<t}^{\left(i\right)})\right]\;. \end{array}$$

Again, we plug Equations (3), (4) and (7) into approximating the integral over $z_{<t}$.

This is the derivation of the entropy term:

$$\begin{array}{cc} \; & -E_{q(z_{1:T}|x_{1:T})}\left[\log q(z_{1:T}|x_{1:T})\right] \end{array}$$

(A12) $$\begin{array}{cc} \; & =-\sum_{t=1}^{T}E_{q(z_{t}|x_{\leq t})}\left[\log q(z_{t}|x_{\leq t})\right] \end{array}$$

(A13) $$\begin{array}{cc} \; & \approx -\sum_{t=1}^{T}\sum_{i=1}^{K}\omega_{t}^{\left(i\right)}E_{q_{\inf}\left(z_{t}|z_{<t}^{\left(i\right)},x_{t}\right)}\left[\log\left(\sum_{i=1}^{K}\omega_{t}^{\left(i\right)}q_{\inf}\left(z_{t}|z_{<t}^{\left(i\right)},x_{t}\right)\right)\right]\;. \end{array}$$

Plugging these all together into Equation (A4), we get the ELBO.

(A14) $$\begin{array}{cc} \log p\left(x_{1:T}\right)\geq L_{\mathrm{ELBO}}\; & =\sum_{t=1}^{T}\sum_{i=1}^{K}\omega_{t}^{\left(i\right)}E_{q_{\inf}\left(z_{t}|z_{<t}^{\left(i\right)},x_{t}\right)}\left[\log p\left(x_{t}|z_{t},z_{<t}^{\left(i\right)}\right)-\log\left(\sum_{i=1}^{K}\omega_{t}^{\left(i\right)}q_{\inf}\left(z_{t}|z_{<t}^{\left(i\right)},x_{t}\right)\right)\right]\\ \; & +E_{q(z_{1}|x_{1})}\left[\log p(z_{1})\right]+\sum_{t=2}^{T}\sum_{i=1}^{K}\omega_{t}^{\left(i\right)}E_{q_{\inf}\left(z_{t}|z_{<t}^{\left(i\right)},x_{t}\right)}\left[\log p(z_{t}|z_{<t}^{\left(i\right)})\right]\;. \end{array}$$

Since $q_{\inf}$ is Gaussian, the expectations are computed with samples. With the weights defined as one hot vectors as in Section 3.4, the computation simplifies further.

## Appendix B. Supplementary to Stochastic Cubature Approximation

The cubature approximation is widely used in the engineering community as a deterministic method to numerically integrate a nonlinear function f(·) of Gaussian random variable $z∼N(\mu_{z},\sigma_{z}^{2}I)$, with $z\in R^{d}$. The method proceeds by constructing 2d+1 sigma points $z^{\left(i\right)}=\mu_{z}+\sigma_{z}\xi^{\left(i\right)}$. The cubature approximation is simply a weighted sum of the sigma points propagated through the nonlinear function f(·),

$$\int f\left(z\right)N\left(z|\mu_{z},\sigma_{z}^{2}I\right)dz\approx \sum_{i=1}^{2d+1}\gamma^{\left(i\right)}f\left(z^{\left(i\right)}\right)\;.$$

Simple analytic formulas determine the computation of weights $\gamma^{\left(i\right)}$ and the locations $\xi^{\left(i\right)}$.

(A15) $$\begin{array}{c} \gamma^{\left(i\right)}=\left\{\begin{array}{cc} \frac{1}{2(n+\kappa )} & ,i=1,\ldots ,2n\\ \frac{\kappa }{n+\kappa } & ,i=0 \end{array}\right.\;\xi^{\left(i\right)}=\left\{\begin{array}{cc} \;\;\;\sqrt{n+\kappa }e_{i} & ,i=1,\ldots ,n\\ -\sqrt{n+\kappa }e_{i-n} & ,i=n+1,\ldots ,2n\\ \;\;\;\;\;\;\;\;\;\;0 & ,i=0\;, \end{array}\right. \end{array}$$

where κ is a hyperparameter controlling the spread of the sigma points in the *n*-dimensional sphere. Further $e_{i}$ represents a basis in the *n*-dimensional space, which is choosen to be a unit vector in cartesian space, e.g., $e_{1}=\left[1,0,\ldots ,0\right]$.

In stochastic cubature approximation (SCA), we adopt the computation of $\xi^{\left(i\right)}$ in Equation (A15), and infuse the sigma points with standard Gaussian noise ϵ∼N(0,I) to obtain stochastic *sigma variables* $s^{\left(i\right)}=\mu_{z}+\sigma_{z}\left(\xi^{\left(i\right)}+\epsilon \right)$. We choose κ=0.5 to set the weights $\gamma^{\left(i\right)}$ equally.

## Appendix C. Supplementary to Experiments Setup

### Appendix C.1. Stochastic Lorenz Attractor Setup

The Lorenz attractor is govern by ordinary differential equations:

$$\frac{dx}{dt}=\sigma \left(y-x\right),\;\frac{dy}{dt}=x\left(\rho -z\right)-y,\;\frac{dz}{dt}=xy-\beta z\;,$$

where σ, ρ, and β are system parameters. We set σ=10,ρ=28 and β=8/3 to make the system chaotic. We simulate the trajectories by RK4 with a step size of 0.01. To make it stochastic, we add noise to the transition, which is a mixture of Gaussians $0.5N\left(m_{0},P\right)+0.5N\left(m_{1},P\right)$, where

$$m_{0}=\left[\begin{array}{c} 0\\ 1\\ 0 \end{array}\right],\;m_{1}=\left[\begin{array}{c} 0\\ -1\\ 0 \end{array}\right],\;P=\left[\begin{array}{ccc} 0.05 & 0.03 & 0.01\\ 0.03 & 0.03 & 0.03\\ 0.01 & 0.03 & 0.05 \end{array}\right].$$

Besides, we add a Gaussian noise with zero mean and diagonal standard deviation [0.6,0.4,0.8] as the observation noise. Totally, we simulate 5000 sequences as a training set, 200 sequences as a validation set, and 800 sequences as a test set. For evaluation of Wasserstein distance, we simulate 10 groups of sequences additionally. Each group has 100 sequences with similar initial observations.

### Appendix C.2. Taxi Trajectories Setup

The full dataset is very large and the length of trajectories varies. We select the trajectories inside the Porto city area with length in the range of 30 and 45, and only extract the first 30 coordinates of each trajectory. Thus, we obtain a dataset with a fixed length of 30. We split it into the training set of size 86,386, the validation set of size 200, and the test set of size 10,000.

### Appendix C.3. U.S. Pollution Data Setup

The U.S. pollution dataset consists of four pollutants (NO_2_, O_3_, SO_2_, and CO). Each of them has 3 major values (mean, max value, and air quality index). It is collected from counties in different states every day from 2000 to 2016. Since the daily measurements are very noisy and volatile, we compute the monthly average values of each measurement, and then extract non-overlapping segments of length 24 from the dataset. In total, we extract 1639 sequences as training set, 25 sequences as validation set, and 300 sequences as test set.

### Appendix C.4. NBA SportVu Data Setup

The dataset consists of sequences of 2D coordinates that describes the movements of basketball players and the ball. We use a sliding window of the width 30, and the stride 30 to cut the long sequences to short sequences of a fixed length 30. We split them into the training set of size 8324, the validation set of size 489, and the test set of size 980.

## Appendix D. Implementation Details

Here, we provide implementation details of VDM models used across the four datasets in the main text. VDM includes:

- Latent RNN: summarize the historic latent states $z_{<t}$ in the hidden states $h_{t}$.
- Transition network: transit the latent states $z_{t}$ temporally.
- Emission network: map the latent states $z_{t}$ and hidden states $h_{t}$ to observations $x_{t}$.
- Inference network: update states $z_{t}$ given observations $x_{t}$ and hidden states $h_{t}$.

The optimizer is Adam with the learning rate of 1 × 10^−3^. In all experiments, the networks have the same architectures but different sizes. The model size depends on observation dimension $d_{x}$, latent state dimension $d_{z}$, and hidden state dimension $d_{h}$. The number of samples used at each time step in the training is $2d_{z}+1$. If the model output is variance, we use a softplus to ensure its non-negative.

- Latent RNN: one layer GRU of input size $d_{z}$ and hidden size $d_{h}$
- Transition network: input size is $d_{h}$; 3 linear layers of size 64, 64, and $2d_{z}$, with ReLUs.
- Emission network: input size is $d_{h}+d_{z}$; 3 linear layers of size 32, 32 and $2d_{x}$, with ReLUs.
- Inference network: input size is $d_{h}+d_{x}$; 3 linear layers of size 64, 64, and $2d_{z}$, with ReLUs.

Here, we give the exact dimension of observations $x_{t}$, latent states $z_{t}$, and hidden states $h_{t}$ of VDM in four experiments in the main text in Table A1. We give the number of parameters for each model in experiments in the main text in Table A2.

**Table A1:** Dimension details of VDM in four experiments.

	$d_{x}$	$d_{z}$	$d_{h}$
Lorenz	3	6	32
Taxi	2	6	32
Pollution	12	8	48
SportVu	2	6	32

**Table A2:** Number of parameters for each model in four experiments. VDM, AESMC, DMM-IAF, VRNN, and RKN have comparable number of parameters. CF-VAE has much more parameters.

	RKN	VRNN	CF-VAE	DMM-IAF	AESMC	VDM
Lorenz	23,170	22,506	7,497,468	24,698	22,218	22,218
Taxi	23,118	22,248	7,491,123	24,536	22,056	22,056
Pollution	35,774	33,192	8,162,850	36,328	31,464	31,464
SportVu	23,118	22,248	7,491,123	24,536	22,056	22,056

All models are trained in our GPU cluster, which consists of NVIDIA GeForce GTX TITAN X GPUs, and NVIDIA TITAN X Pascal GPUs. Since VDM has a small model size, the performance does not rely on the hardware and the training has no high hardware requirements.
